# High mortality among patients with cancer and COVID-19 infection: the experience of a Brazilian cancer center

**DOI:** 10.31744/einstein_journal/2021AO6254

**Published:** 2021-10-26

**Authors:** Lilian Arruda do Rêgo Barros, Marcos Aurélio Fonseca Magalhães, Rafaela de Brito Alves, Camilla Vieira de Rebouças, Cicilia Marques Rodrigues, Micaela Mazutti Viu, Vinicius de Lima Benedito, Alayne Magalhães Trindade Domingues Yamada, Auro del Giglio, Felipe José Silva Melo Cruz

**Affiliations:** 1 Instituto Brasileiro de Controle do Câncer São PauloSP Brazil Instituto Brasileiro de Controle do Câncer, São Paulo, SP, Brazil.; 2 Centro Universitário FMABC Santo AndréSP Brazil Centro Universitário FMABC, Santo André, SP, Brazil.; 3 Centro Universitário São Camilo São PauloSP Brazil Centro Universitário São Camilo, São Paulo, SP, Brazil.

**Keywords:** Neoplasms, COVID-19, SARS-CoV-2, Coronavirus infections, Mortality, Pandemics, Fatal outcome

## Abstract

**Objective:**

To evaluate the severity of COVID-19 in cancer patients to describe clinical and epidemiological factors associated with poor outcomes (mortality and need of intensive care unit admission or mechanical ventilation).

**Methods:**

Retrospective data from patients with cancer and laboratory diagnosis of COVID-19, obtained between March 16 and May 29, 2020, were retrieved out of a cancer center database. Data analyzed included patient history, age, sex, comorbidities, types of cancer and anticancer therapy.

**Results:**

This sample comprised 105 patients aged 18-92 years, 80.9% of whom were females. Dyspnea was the most prevalent initial symptom (30.4%) among patients who died (p<0.0001). Overall, 57.1% of patients had metastatic disease and 60% had poor performance status (Eastern Cooperative Oncologic Group ≥2) at the time of COVID-19 diagnosis. The overall mortality rate was 40.95%. Mortality rates were higher in male patients and those with poor performance status (p<0.0001).

**Conclusion:**

This cohort is one of the largest Brazilian studies describing clinical and epidemiological features of patients with cancer and concurrent COVID-19. Findings of this study emphasize the vulnerability of cancer patients in the current pandemic, and indicate high mortality from COVID-19 among male cancer patients and cancer patients with poor performance status. This analysis may assist the selection of patients who may benefit from strict isolation and eventual discontinuation of anticancer therapy to reduce exposure to infection.

## INTRODUCTION

The first case of coronavirus disease 2019 (COVID-19) was reported on December 31, 2019.^(1)^ The genetic sequence of the novel coronavirus was isolated from patients presenting with respiratory infection in Wuhan, China, on January 7, 2020.^(2)^ This new disease became a global pandemic in a matter of weeks. By the end of March 2020, COVID-19 had caused 40,673 deaths, affecting 824,559 people in more than one hundred countries.^(1)^ At this stage, the five countries with the largest cumulative number of cases are China, Italy, United States, Spain and Germany (24.6%, 17.8%, 9.5%, 8.6% and 7.5% reported cases, respectively), and the countries with highest mortality rates being Italy (9.3%), Iran (7.8%) and Spain (6%).^(1)^

In Latin America and the Caribbean, approximately 21% of population is estimated to be at risk of developing severe COVID-19 disease. This region has experienced major outbreaks, with a growing number of cases in Brazil, Peru, Mexico, Chile, Colombia, Panama (and possibly Venezuela and Nicaragua), and increasing concerns regarding testing capacity for COVID-19.^(3-6)^

Although the COVID-19 epidemic continues to grow in Brazil, the transmission potential, clinical and epidemiological characteristics of this disease are still poorly understood. The transmissibility of severe acute respiratory syndrome coronavirus 2 (SARS-CoV-2) is thought to be higher in Brazil than in Italy, United Kingdom, France, and Spain. As in other densely populated countries, COVID-19 spread rapidly throughout the Brazil. Cities with greater connectivity with other regions of the country, or other countries, were affected sooner than less densely populated municipalities. In the initial phase of the COVID-19 pandemic in Brazil, higher rates of confirmed COVID-19 cases were reported in the metropolitan region of São Paulo, (SP), especially in census sectors with higher income *per capita*.^(7)^ São Paulo, (SP) is the largest city in South America and the most common destination of international flights in Brazil. According to the World Health Organization (WHO), Brazil currently has one of the fastest growing COVID-19 rates worldwide, with 1,864,681 cases and 72,100 deaths, and accounts for more than 55% of total number of reported cases in Latin America and the Caribbean (data from July 14, 2020).^(8)^

Health conditions associated with increased risk of poor clinical COVID-19 prognosis have been well described and include hypertension, chronic obstructive pulmonary disease, *diabetes mellitus*, cardiovascular diseases, and immunosuppressive disorders. For example, patients with active cancer are often immunocompromised and hence more prone to severe COVID-19.^(9,10)^ In a prospective cohort study with COVID-19 patients, patients who had recently been submitted to cancer treatment had a higher risk of severe events compared to untreated patients. In fact, that study was the first to report higher risks of severe events in patients with active cancer relative to patients with stable disease. Cancer treatments described in that cohort were curative surgical treatment (tumor resection) or chemotherapy.^(11)^

Increased risk of mortality due to COVID-19 in cancer patients has been suggested. Therefore, oncology societies around the world, such as the European Society of Medical Oncology (ESMO), the American Society of Clinical Oncology (ASCO), the National Comprehensive Cancer Network^®^ (NCCN^®^) and others, have developed guidelines aimed to mitigate the negative impacts of the COVID-19 pandemic on cancer diagnosis and treatment.^(12)^ Categorization of patients as (high, medium, or low) priority for treatment planning purposes is a common feature to these guidelines. In the current evolving scenario, pragmatic approaches are needed to address the challenges involved in cancer patient treatment, not hindering their care.^(13)^ Hospitals worldwide have also published internal guidelines for oncologists, to reduce patient exposure to COVID-19. In view of the immunocompromised nature of these patients, cancer centers have adhered to strict infection control guidelines, in both inpatient and outpatient settings, and have implemented similar guidelines for outpatient visits, including reduction of outpatient and chemotherapy infusion visits.^(14)^

Outcomes of cancer patients affected with COVID-19 have been examined in a prior meta-analysis of 26 studies, involving 181,323 patients overall and 23,736 oncologic patients.^(12)^ That analysis revealed a higher risk of death in cancer patients with COVID-19. Cancer patients were also more likely to be intubated, in spite of similar rates of intensive care unit (ICU) admission. With regard to cancer subtypes, mortality was highest in cases of hematologic neoplasms, followed by lung cancer. However, no associations between type of cancer therapy and mortality were found.^(12)^

Robust studies revealed that cancer patients with COVID-19 have higher morbidity and mortality rates and their appropriate management requires a deeper understanding of interactions between therapies targeting cancer and COVID-19.^(12)^ Given the small number of publications addressing management and survival of cancer patients with COVID-19, epidemiological and clinical data on the impact of COVID-19 on this patient population are still scarce.

## OBJECTIVE

To evaluate the severity of COVID-19 in cancer patients and to describe clinical and epidemiological features associated with poor outcomes (mortality and need for intensive care unit admission or mechanical ventilation).

## METHODS

This is a retrospective single-center cohort study with adult cancer patients routinely followed up at *Instituto Brasileiro de Controle do Câncer* (IBCC Oncologia, São Paulo, SP, Brazil). Consecutive patients diagnosed with COVID-19 between March 16 and May 29, 2020 were included in the study. This study was approved by the Brazilian National Research Ethics Committee (Conep - *Comissão Nacional de Ética em Pesquisa*), protocol 3.968.710, CAAE: 30579720.4.0000.0008, and granted waiver of informed consent due to observational design. Patients were enrolled at the time of COVID-19 diagnosis and followed for up to 30 days. COVID-19 diagnosis was achieved using established methods (reverse transcription polymerase chain reaction – RT-PCR – testing), of nasopharyngeal swabs or sputum specimens.

Patients in this sample had symptoms of COVID-19 at the time of RT-PCR testing due to the shortage of laboratory tests in Brazil at that time. Patients with benign or non-invasive malignant neoplasms were excluded.

Clinical characteristics, laboratory and radiological changes, COVID-19 treatment and disease outcome data were extracted from IBCC Oncologia medical records using a dedicated data collection form. Imaging and laboratory tests were performed using standard methodology. Laboratory tests consisted of complete blood count, liver and kidney function tests, coagulation tests and procalcitonin, electrolyte, C-reactive protein, D-dimer and lactate dehydrogenase level determination. Outpatients and patients discharged from hospital were followed by telephone call for 30 days after the COVID-19 diagnosis.

The primary endpoint in this study was a composite of ICU admission, need of mechanical ventilation or death. Secondary endpoints were death rates and time from onset of symptoms to primary composite endpoint.

### Statistical analysis

Continuous variables in this report were expressed as medians and interquartile or simple intervals, as appropriate. Categorical variables were summarized as counts and percentages. Missing data were not imputed.

Proportions of nominal categorical variables were compared using the χ^2^ test. Continuous variables were compared using the Student *t*-test. Factors associated with the primary composite outcome were analyzed using binary logistic regression. P values <0.05 were considered significant.

Descriptive and multiple logistic regression analyses were carried out using death as the outcome variable. Exploratory analysis of selected patients based on performance status according to Eastern Cooperative Oncologic Group (ECOG) score was also carried out. No adjustments were made for multiple testing due to the small number of events. Statistical analyses were performed using software STATA 16.

## RESULTS

In this retrospective single-center cohort study, the analyses carried out on July 5, 2020 included data from 105 cancer patients diagnosed as COVID 19, at IBCC Oncologia, between March 16 and May 29, 2020.

### Clinical characteristics of patients

Baseline characteristics of patients in this sample at the time of COVID-19 diagnosis are shown in table 1. The study population comprised 85 females (80.9%). The median age at diagnosis of patients who died was significantly higher relative to patients who survived (61 [18-92] years and 49 [25-85] years, respectively; p=0.013).

**Table 1:** Clinical characteristics of patients

Characteristics	Alive (n=63)n (%)	Deceased (n=42) n (%)	p value
Median age (range)	49 (25-85)	61 (18-92)	0.013*
Male	4 (20)	16 (80)	<0.001*
Female	58 (68.2)	27 (31.8)	<0.001*
Chronic lung disease	2 (1.9)	3 (2.9)	0.07*
Comorbidities			
DM	12 (11.4)	10 (9.5)	0.23*
Hypertension	22 (20.9)	18 (17.1)	0.44*
CHF	3 (2.9)	4 (3.8)	0.81*
Symptoms			
Fever	20 (19)	12 (11.4)	0.63^†^
Cough	26 (24.7)	14 (13.3)	0.33^†^
Dyspnea	16 (15.2)	32 (30.4)	<0.001^†^
Fatigue	10 (9.5)	4 (3.8)	0.31^†^
Myalgia	13 (12.3)	1 (0.9)	0.007^†^
Anosmia	6 (5.7)	0	0.07^†^
Solid tumors	61 (58)	39 (37.1)	0.08*
Breast	27 (25.7)	11 (10.4)	
Genitourinary	2 (1.9)	5 (4.7)	
Gastrointestinal	5 (4.7)	4 (3.8)	
Gynecologic	23 (21.9)	12 (11.4)	
Lung	3 (2.8)	0	
Skin, non-melanoma	0	3 (2.8)	
Head and neck	1 (0.9)	4 (3.8)	
Hematologic malignancies	1 (0.9)	4 (3.8)	
Metastatic disease			0.005*
No	34 (32.3)	11 (10.4)	
Yes	28 (28.6)	32 (30.4)	
Performance status (ECOG)			0.0001*
0-1	35 (33.6)	7 (6.7)	
2-4	27 (25.9)	35 (33.6)	
Do-not-resuscitate			0.048*
No	54 (51.4)	31 (29.5)	
Yes	8 (7.6)	12 (11.4)	
Active chemotherapy			0.518*
No	45 (42.8)	28 (26.6)	
Yes	17 (16.1)	15 (14.2)	
Active radiotherapy			0.714*
No	58 (55.2)	39 (37.1)	
Yes	4 (3.8)	4 (3.81)	
Recent surgery			0.661*
No	46 (43.8)	30 (28.5)	
Yes	16 (15.2)	13 (12.3)	

Age stated as median and range. Other variables stated as absolute numbers and percent.

* χ^2^ test; ^†^ Student’s *t* test.

DM: *diabetes mellitus*; CHF: congestive heart failure; ECOG: Eastern Cooperative Oncology Group.

Breast cancer was the most frequent type of cancer, followed by gynecologic and gastrointestinal cancer (38/36.1%, 35/33.3% and 9/8.57% affected patients, respectively). Sixty patients (57%) had stage IV disease, and 40% had good ECOG performance status (ECOG 0-1), at the time of COVID-19 diagnosis.

Out of 60 patients (30.4%) undergoing active anticancer therapy, 42 (70%) received cytotoxic chemotherapy, six (10%) target agents and 12 (20%) endocrine therapy. Patients in this sample did not receive immune checkpoint inhibition therapy. Eight patients (7.6%) were undergoing radiation therapy, and 29 patients had recently been submitted to surgery (*i.e*., operated on 30 days or less prior to the onset of COVID-19 symptoms). Active cancer treatment (surgery, radiation therapy or systemic treatment) was not associated with mortality.

Major comorbidities in cancer patients with COVID-19 included hypertension (38%), diabetes (20.9%) and heart disease (6.7%). Dyspnea, cough, and fever were the most common symptoms upon COVID-19 diagnosis, observed in 45.6%, 38% and 30.4% of patients, respectively. Other symptoms included fatigue (13.3%), myalgia (13.2%) and anosmia (5.7%).

### Clinical measures and outcomes of cancer patients with COVID-19

As of July 5, 2020, 49 out of 105 (46.6%) cancer patients with COVID-19 were considered to recovered or cured (mean follow-up of 30 days). Forty-three patients (40.92%) had died at the time of the last follow-up (Table 2).

**Table 2:** Clinical measures and outcomes of cancer patients with COVID-19

Variable	Total (%)	Alive (%)	Deceased (%)	p value
Hospital admission, n (%)	73 (69.5)	30 (28.5)	43 (40.9)	0.001
Hospital length of stay, days	17.9±12.9	14.7±11.7	20.1±13.3	0.03
ICU admission, n (%)	35 (33)	4 (3.8)	31 (29.5)	0.001
ICU length of stay, days	4.7±6.8	0.5±2.2	8.4±7.4	0.001
Mechanical ventilation, n (%)	34 (32.3)	2 (1.9)	32 (30.4)	0.001
Disseminated intravascular coagulation, n (%)	3 (2.86)	0	3 (2.86)	0.001
Pneumonia, n (%)	49 (46.6)	14 (13.3)	35 (33.3)	0.001
Renal replacement therapy, n (%)	25 (23.8)	3 (2.8)	22 (20.9)	0.001
Septic shock, n (%)	31 (29.5)	1 (0.9)	30 (28.5)	0.001

Results expressed as absolute numbers and percent (%) or mean±standard deviation.

ICU: intensive care unit.

Seventy-three patients (69.5%) required hospital admission due to COVID-19 disease, whereas 32 (30.4%) were discharged home. Hospital length of stay ranged from 12.9 to 17.9 days.

The mortality rate of patients requiring hospital admission was 40.95%, with higher rates among male patients (only 20% survival at the study cutoff date, compared to 68.2% survival among female patients). After hospital admission, 35 patients (33.3%) required ICU admission. Only four out of 35 patients requiring ICU admission were alive at the last follow-up.

Hospitalized patients were submitted to laboratory tests (complete blood count, serum biochemistry, coagulation test, liver and kidney function tests, electrolytes, C-reactive protein, procalcitonin, D-dimer, and lactate dehydrogenase level determination). Laboratory markers were not predictors of higher risk of mortality.

In this study, patients requiring mechanical ventilation or renal replacement therapy tended to have high death rates (32 out of 34 patients/94.1%, and 22 out of 25/88%, respectively). Dyspnea was the most prevalent initial symptom among patients who died (30.4%) and was significantly (p<0.0001) associated with this outcome.

Hospital admission, need for ICU admission, and longer ICU stay had a significant (p<0.0001) negative impact on patient outcome, whereas hospital length of stay did not (p=0.03).

Five cases of arrhythmia (one right bundle branch block and four atrial fibrillations with high ventricular response), and three microvascular thrombotic events (disseminated intravascular coagulation) were identified in this sample. Patients who developed arrhythmia or disseminated intravascular coagulation progressed to death. No dermatological events were recorded, since associations between such events and COVID-19 had already been recognized when the registry was created.

### Logistic regression analysis and odds of dying according to clinical characteristics of cancer patients

In this study, poor performance status (odds ratio – OR of 5.15; 95% confidence interval – 95%CI: 1.15-22.97; p=0.031) and male sex (OR of 7.29; 95%CI: 1.01-52.33; p=0.048) were associated with increased risk of death (multiple logistic regression analysis, table 3). Do-not-resuscitate order and stage IV disease (presence of metastasis) were not associated with higher mortality.

**Table 3:** Logistic regression analysis and odds of dying according to clinical characteristics of cancer patients

Characteristics	OR (95%CI)	p value
Sex	7.29 (1.01-52.33)	0.048
Age	1.01 (0.96-1.06)	0.594
Performance status	5.15 (1.15-22.97)	0.031
Do-not-resuscitate	0.48 (0.08-2.82)	0.419
C-reactive protein (mg/dL)	1.02 (0.95-1.09)	0.482
Stage IV disease	3.06 (0.66-19.25)	0.139
Number of comorbidities	0.77 (0.30-1.95)	0.590

OR: odds ratio; 95%CI: 95% confidence interval.

### Exploratory analysis of patients according to performance status

On top of factors listed in table 4, exploratory analysis according to performance status of this population of cancer patients with COVID-19 revealed significant (p<0.0001) associations between COVID-19 severity and ICU admission, need of mechanical ventilation, and longer ICU stay, but not with hospital length of stay (p=0.059).

**Table 4:** Exploratory analysis of patients according to performance status

Clinical outcomes	ECOG 0-1 (n=42)	ECOG 2-4 (n=62)	p value
Hospital admission required, n (%)	18 (17.3)	54 (51.9)	<0.001
Hospital length of stay, days	11±6.9	19.9±13.6	0.059
ICU admission, n (%)	7 (6.3)	27 (25.9)	0.005
Length of ICU stay, days	2.0±3.9	5.56±7.32	0.007
Mechanical ventilation, n (%)	7 (6.3)	26 (25)	0.005
Death, n (%)	7 (6.73)	35 (33.6)	<0.001

Results expressed as absolute numbers and percent (%) or mean±standard deviation.

ECOG: Eastem Cooperative Oncologic Group; SD: standard deviation; ICU: intensive care unit.

## DISCUSSION

The COVID-19 outbreak became a viral pandemic feared by cancer patients and oncology teams due to the significant negative impacts on cancer treatment.^(15)^ This report describes clinical and epidemiologic features of cancer patients with COVID-19 seen at a Brazilian cancer center.

*Instituto Brasileiro de Controle do Câncer Oncologia* is a referral oncology center in Brazil. Approximately 15 thousand patients were actively treated at IBCC Oncologia in the 3 months prior to the COVID-19 outbreak. Of these, only 105 were diagnosed with SARS-CoV-2 infection, between March 16 and May 29, 2020. An estimated 170,342 people living in urban São Paulo, (SP) had been infected with the SARSCoV-2 up to the date of this report.^(16)^

The incidence of COVID-19 in cancer patients cannot be determined based on this study. The apparently low incidence may have reflected the adoption of preventive measures, such as social distancing by these patients, who had been duly informed about their potentially higher risk of severe COVID-19, or prophylactic measures implemented at IBCC Oncologia to ensure a safe flow of patients within the hospital, as proposed by several guidelines.^(17-21)^

Breast and gynecologic cancer were the most common type of neoplasms in this cohort. Similar findings have been reported in previous studies describing relations between type of cancer and COVID-19.^(22)^ Different from other studies, higher prevalence of lung cancer was not observed in this sample.^(23)^ This discrepancy may be explained by the fact that IBCC Oncologia specializes in women’s cancers.

Typical symptoms of COVID-19 in cancer patients in this study (fever, cough, dyspnea, fatigue, myalgia and anosmia) did not differ from those reported in the general population.^(24)^

In this cohort, dyspnea was the first symptom experienced by patients who died (30.4%), and significant (p<0.0001) associations between both events were found. This finding indicates dyspnea should be given special attention in management of cancer patients with COVID-19.

According to previous studies, dyspnea occurs at a much earlier stage, right from the onset of COVID-19, in lung cancer patients relative to the general population and patients suffering from other cancers. Patients with lung cancer have poorer baseline lung function and resistance, are more likely to develop severe anoxia and experience faster progression of COVID-19.^(24,25)^ These factors could not be analyzed in this study due to the low number of cases of lung cancer (two) in the sample.

The mortality rate of patients requiring hospital admission in this cohort (40.95%) is consistent with findings of a systematic review of 52 studies and 18,650 cancer patients with COVID-19, a clear indication that mortality is in fact higher in these patients, and hospital admission should be considered an independent risk factor.^(26)^ Two independent risk factors for mortality were identified in logistic regression analysis in this study: male sex and ECOG performance status of 2 or higher. Higher mortality among male patients may have reflected the small number of males in this sample. Also, given the small sample size, poor performance status may have outweighed other risk factors.

In contrast with findings of this report, several other studies conducted throughout these months of pandemic failed to reveal higher mortality rates among cancer patients with COVID-19.^(27-40)^

Data presented in this report suggest clinicians should approach cancer patients with ECOG performance status of two or higher as an extremely vulnerable population, which should be a major target of social isolation measures.

A Brazilian cohort study with cancer patients with COVID-19, conducted by de Melo et al., and published in October 2020, revealed similar findings. In that study, the COVID-19-specific mortality rate of patients with solid tumors was 37.7%, and rates of complications, such as respiratory failure, septic shock and acute kidney injury were significantly high (38.7%, 22.1% and 18.2%, respectively).^(41)^

This study has some limitations. First, at the beginning of the COVID-19 pandemic, patients sought medical care only when experiencing severe symptoms, such as dyspnea, persistent fever, or chest pain. Therefore, severe cases of COVID-19 may have been overrepresented, which may partially explain the high hospital admission rate (70%) in this sample. Second, patients with mild COVID-19 symptoms may have chosen to stay at home or sought Care at Primary Health units.

This analysis was based on the first COVID-19 cases recorded at IBCC Oncologia. Future studies are warranted, given the currently growing number of cases.

## CONCLUSION

In spite of adopting preventive social distancing measures by cancer patients, and prophylactic measures implemented at *Instituto Brasileiro de Controle do Câncer* to ensure a safe flow of patients within the hospital, this analysis revealed high COVID-19 mortality rates in male cancer patients, and cancer patients with poor performance status.

These findings emphasize the vulnerability of cancer patients in the current pandemic scenario, and may contribute to more appropriate selection of patients, who may benefit from strict isolation and eventual discontinuation of anticancer treatment to reduce exposure to COVID-19 infection.
