# Supplementary material for: High mortality among patients with cancer and COVID-19 infection: the experience of a Brazilian cancer center
Source: Einstein (Sao Paulo). 2021 Oct 26;19:eAO6254. doi: 10.31744/einstein_journal/2021AO6254 (PMC8555874; doi:10.31744/einstein_journal/2021AO6254)
Supplement: Translation

# Translation: Alta mortalidade entre pacientes com câncer e infecção por COVID-19: a experiência de um centro oncológico brasileiro

**Authors:** Lilian Arruda do Rêgo Barros, Marcos Aurélio Fonseca Magalhães, Rafaela de Brito Alves, Camilla Vieira de Rebouças, Cicilia Marques Rodrigues, Micaela Mazutti Viu, Vinicius de Lima Benedito, Alayne Magalhães Trindade Domingues Yamada, Auro del Giglio, Felipe José Silva Melo Cruz

**Affiliations:** 1 Brasil Instituto Brasileiro de Controle do Câncer, São Paulo, SP, Brasil.; 2 Brasil Centro Universitário FMABC, Santo André, SP, Brasil.; 3 Brasil Centro Universitário São Camilo, São Paulo, SP, Brasil.

**Keywords:** Neoplasias, COVID-19, SARS-CoV-2, Infecções por coronavírus, Mortalidade, Pandemias, Evolução fatal

## Abstract

**Objetivo:**

Avaliar a gravidade da infecção por COVID-19 em pacientes oncológicos, determinando os aspectos clínicos e epidemiológicos associados ao pior desfecho, seja em termos de mortalidade, necessidade de internação em unidade de terapia intensiva ou ventilação mecânica.

**Métodos:**

Pacientes com câncer e diagnóstico confirmado por laboratório de COVID-19 foram identificados nos bancos de dados de um hospital oncológico entre 16 de março e 29 de maio de 2020. Os dados coletados incluíram história, idade, sexo e comorbidades dos pacientes, além dos tipos de câncer e do tratamento anticâncer.

**Resultados:**

Dentre os 105 pacientes analisados, a idade variou de 18 a 92 anos, e 80,9% eram do sexo feminino. Dispneia foi o sintoma inicial mais prevalente entre os que morreram (30,4%). No momento do diagnóstico da infecção, 57,1% apresentavam doença metastática e 60% *performance status* ruim (*Eastern Cooperative Oncologic Group* ≥2). A taxa de mortalidade geral foi 40,95% e superior entre os homens e pacientes com baixo nível de *performance status* (p<0,0001).

**Conclusão:**

Este coorte é um dos estudos mais robustos do Brasil, descrevendo características clínicas e epidemiológicas de pacientes com câncer e COVID-19. Os achados do estudo alertam para a vulnerabilidade dos pacientes oncológicos na pandemia atual e demonstram alta mortalidade por COVID-19 em pacientes do sexo masculino e com pior *performance status*. Essa análise pode ajudar a selecionar os pacientes que podem se beneficiar de isolamento rigoroso e até mesmo da interrupção do tratamento, reduzindo a exposição à infecção.

## INTRODUÇÃO

O primeiro caso da doença causada pelo coronavírus 2019 (COVID-19) foi relatado em 31 de dezembro de 2019.^(1)^ A sequência genética do novo coronavírus foi isolada de pacientes que apresentaram infecção respiratória em Wuhan, na China, em 7 de janeiro de 2020.^(2)^ Em questão de semanas, essa nova doença se tornou uma pandemia global. Ao final de março de 2020, a COVID-19 tinha causado 40.673 mortes, afetando 824.559 pessoas, em mais de cem países.^(1)^ Atualmente, os cinco países com maior número cumulativo de casos são China, Itália, Estados Unidos, Espanha e Alemanha (24,6%, 17,8%, 9,5%, 8,6% e 7,5% casos relatados, respectivamente), sendo a Itália (9,3%), o Irã (7,8%) e a Espanha (6%) os com as taxas mais elevadas de mortalidade.^(1)^

Na América Latina e no Caribe, acredita-se que aproximadamente 21% da população tenha risco de apresentar a forma grave de COVID-19. Nessa região houve grandes surtos, com número crescente de casos no Brasil, no Peru, no México, no Chile, na Colômbia e no Panamá (e, possivelmente, na Venezuela e na Nicarágua), além de preocupações crescentes a respeito da capacidade de testagem para COVID-19.^(3-6)^

Embora a pandemia de COVID-19 continue a evoluir no Brasil, os detalhes concernentes ao potencial de transmissão e às características clínicas e epidemiológicas da doença ainda não foram totalmente elucidados. Acredita-se que o potencial de transmissão do coronavírus da síndrome respiratória aguda grave 2 (SARS-CoV-2) no Brasil seja mais alto do que na Itália, no Reino Unido, na França e na Espanha. Assim como em outros países densamente povoados, a COVID-19 se disseminou rapidamente em todo o território nacional. As cidades com maior grau de conexão com outras regiões do país ou outros países foram afetadas antes de municipalidades menos populosas. Na fase inicial da pandemia de COVID-19 no Brasil, taxas elevadas de casos confirmados de COVID-19 foram relatadas na região metropolitana de São Paulo (SP), sobretudo em setores censitários com renda *per capita* mais alta.^(7)^ São Paulo, (SP) é a maior cidade da América do Sul e o destino mais comum de voos internacionais que chegam ao Brasil. Segundo a Organização Mundial da Saúde (OMS), o Brasil tem, atualmente, uma das taxas de COVID-19 que mais crescem no mundo, com 1.864.681 casos e 72.100 mortes, respondendo por mais de 55% do número total de casos na América Latina e no Caribe (dados de 14 de julho de 2020).^(8)^

As doenças associadas a maior risco de COVID-19 com mau prognóstico clínico foram bem descritas e incluem hipertensão, doença pulmonar obstrutiva crônica, *diabetes mellitus*, doenças cardiovasculares e condições associadas à imunossupressão. Por exemplo, pacientes com câncer ativo costumam ser imunocomprometidos e, então, mais predispostos a quadros graves de COVID-19.^(9,10)^ Em um estudo prospectivo de coorte com pacientes com câncer e COVID-19, o risco de eventos graves foi mais alto entre aqueles que tinham sido submetidos a tratamento antineoplásico recente do que entre os não tratados. Na verdade, o estudo em questão foi o primeiro a relatar alto risco de eventos graves em pacientes com câncer ativo em relação àqueles com doença estável. Os tratamentos antineoplásicos descritos foram cirurgia curativa (ressecção do tumor) ou quimioterapia.^(11)^

Foi sugerido que o risco de mortalidade por COVID-19 é maior em pacientes com câncer. Por esse motivo, diversas sociedades de oncologia, como a *European Society of Medical Oncology* (ESMO), a *American Society of Clinical Oncology* (ASCO) e a *National Comprehensive Cancer Network*^*®*^ (NCCN^®^), entre outras, elaboraram diretrizes destinadas a mitigar os impactos negativos da pandemia de COVID-19 no diagnóstico e no tratamento de pacientes com câncer.^(12)^ A classificação desses pacientes em termos de prioridade (alta, média ou baixa) de tratamento é um ponto comum a todas essas diretrizes. Dada a situação vigente, abordagens pragmáticas se fazem necessárias para o enfretamento dos desafios envolvidos no tratamento de pacientes com câncer, sem prejuízo dos cuidados a eles destinados.^(13)^ Hospitais no mundo todo também publicaram diretrizes internas para oncologistas, com o intuito de reduzir a exposição dos pacientes à COVID-19. Em vista do comprometimento imunológico desses pacientes, os centros de tratamento de câncer adotaram regras rígidas de controle da infeção, tanto nos setores de internação quanto nos ambulatoriais, e implementaram diretrizes semelhantes para atendimento ambulatorial, incluindo a restrição do número de consultas e comparecimentos para infusão de quimioterapia.^(14)^

Os desfechos de pacientes com câncer afetados por COVID-19 foram analisados em metanálise prévia de 26 estudos, que envolveu um total de 181.323 pacientes e 23.736 pacientes oncológicos.^(12)^ Essa análise revelou um risco mais elevado de morte em pacientes com câncer e COVID-19. A probabilidade de intubação também foi maior entre os pacientes com câncer, embora o índice de internação em unidade de terapia intensiva (UTI) não tenha diferido. No que se refere a subtipos de câncer, a mortalidade foi maior em casos de neoplasias hematológicas, seguidas do câncer pulmonar. Entretanto, não foram detectadas associações entre tipo de terapia antineoplásica e mortalidade.^(12)^

Estudos robustos revelaram que pacientes com câncer e COVID-19 apresentam taxas mais altas de morbidade e mortalidade, e seu manejo adequado requer uma compreensão mais profunda das interações entre as terapias voltadas para o tratamento do câncer e para a COVID-19.^(12)^ Dado o pequeno número de publicações que abordaram o tratamento e a sobrevida de pacientes com câncer e COVID-19, os dados epidemiológicos e clínicos referentes ao impacto da COVID-19 nessa população de pacientes ainda são escassos.

## OBJETIVO

Avaliar a gravidade da COVID-19 em pacientes com câncer e descrever as características clínicas e epidemiológicas associadas a desfechos desfavoráveis (mortalidade, necessidade de internação em unidade de terapia intensiva e ventilação mecânica).

## MÉTODOS

Trata-se de um estudo retrospectivo unicêntrico de coorte realizado com pacientes adultos acompanhados rotineiramente no Instituto Brasileiro de Controle do Câncer (IBCC Oncologia, São Paulo, SP, Brasil). Pacientes consecutivos diagnosticados com COVID-19 entre 16 de março e 29 de maio de 2020 foram incluídos no estudo. Este estudo foi aprovado pela Comissão Nacional de Ética em Pesquisa (Conep), protocolo n^o^ 3.968.710, CAAE: 30579720.4.0000.0008, e isento de consentimento informado devido ao desenho experimental observacional. Os pacientes foram incluídos por ocasião do diagnóstico de COVID-19 e acompanhados por até 30 dias. O diagnóstico de COVID-19 foi obtido por meio de métodos consagrados (reação em cadeia da polimerase via transcriptase reversa – RT-PCR), a partir de *swabs* nasofaríngeos ou amostras de escarro.

Os pacientes incluídos nesta amostra apresentavam sintomas de COVID-19 ao serem submetidos à testagem por RT-PCR, devido à escassez de testes laboratoriais no Brasil na época. Pacientes com neoplasias benignas ou malignas não invasivas foram excluídos.

Os dados sobre características clínicas, alterações laboratoriais e de imagem, tratamento para COVID-19 e desfecho da doença foram extraídos dos prontuários médicos do IBCC Oncologia, empregando-se um formulário específico para esse fim. Os exames de imagem e laboratoriais foram realizados empregando-se a metodologia padrão. Os exames laboratoriais incluíram hemograma, testes de função renal e hepática, testes de coagulação, procalcitonina, dosagens de eletrólitos, proteína C-reativa, D-dímero e lactato desidrogenase. Os pacientes ambulatoriais e os que foram liberados foram acompanhados por ligação telefônica por 30 dias a partir da data do diagnóstico.

Neste estudo, o desfecho primário composto incluiu internação na UTI, necessidade de ventilação mecânica e morte. Os desfechos secundários foram as taxas de mortalidade e o tempo decorrido do início dos sintomas até o desfecho primário composto.

### Análise estatística

Neste relato, as variáveis contínuas foram expressas na forma de medianas e intervalos simples ou interquartis, conforme apropriado. As variáveis categóricas foram resumidas como contagens e percentagens. Os dados faltantes não foram computados.

As proporções de variáveis categóricas nominais foram comparadas empegando-se o teste do χ^2^. As variáveis contínuas foram comparadas empregando-se o teste *t* de Student. Os fatores associados ao desfecho primário composto foram analisados empregando-se a regressão logística binária. Valores de p<0,05 foram considerados significativos.

As análises descritiva e de regressão logística múltipla foram realizadas adotando-se morte como variável de desfecho. Realizou-se também a análise exploratória dos pacientes selecionados, de acordo com o escore *performance status* do *Eastern Cooperative Oncologic Group* (ECOG). Em vista do número limitado de eventos, não foram realizados ajustes nos testes múltiplos. As análises estatísticas foram realizadas empregando-se o *software* STATA 16.

## RESULTADOS

Neste estudo retrospectivo unicêntrico de coorte, as análises realizadas em 5 de julho de 2020 foram baseadas nos dados de 105 pacientes com câncer diagnosticados com COVID-19 no IBCC Oncologia, entre 16 de março e 29 de maio de 2020.

### Características clínicas dos pacientes

As características iniciais dos pacientes incluídos nesta amostra no momento do diagnóstico de COVID-19 encontram-se dispostas na tabela 1. A população do estudo foi composta de 85 mulheres (80,9%). A mediana de idade dos pacientes que faleceram foi significativamente mais alta dos que a dos que sobreviveram (61 [18-92] anos e 49 [25-85] anos, respectivamente; p=0,013).

**Tabela 1:** Características clínicas dos pacientes

Características	Vivos (n=63) n (%)	Falecidos (n=42) n (%)	Valor de p
Mediana de idade	49 (25-85)	61 (18-92)	0,013*
Homens	4 (20)	16 (80)	<0,001*
Mulheres	58 (68,2)	27 (31,8)	<0,001*
Doença pulmonar crônica	2 (1,9)	3 (2,9)	0,07*
Comorbidades			
DM	12 (11,4)	10 (9,5)	0,23*
Hipertensão	22 (20,9)	18 (17,1)	0,44*
ICC	3 (2,9)	4 (3,8)	0,81*
Sintomas			
Febre	20 (19)	12 (11,4)	0,63^†^
Tosse	26 (24,7)	14 (13,3)	0,33^†^
Dispneia	16 (15,2)	32 (30,4)	<0,001^†^
Fadiga	10 (9,5)	4 (3,8)	0,31^†^
Mialgia	13 (12,3)	1 (0,9)	0,007^†^
Anosmia	6 (5,7)	0	0,07^†^
Tumores sólidos	61 (58)	39 (37,1)	0,08*
Mama	27 (25,7)	11 (10,4)	
Geniturinário	2 (1,9)	5 (4,7)	
Gastrintestinal	5 (4,7)	4 (3,8)	
Ginecológico	23 (21,9)	12 (11,4)	
Pulmonar	3 (2,8)	0	
Pele, não melanoma	0	3 (2,8)	
Cabeça e pescoço	1 (0,9)	4 (3,8)	
Malignidades hematológicas	1 (0,9)	4 (3,8)	
Doença metastática			0,005*
Não	34 (32,3)	11 (10,4)	
Sim	28 (28,6)	32 (30,4)	
*Performance status* (ECOG)			0,0001*
0-1	35 (33,6)	7 (6,7)	
2-4	27 (25,9)	35 (33,6)	
Ordem para não ressuscitação			0,048*
Não	54 (51,4)	31 (29,5)	
Sim	8 (7,6)	12 (11,4)	
Quimioterapia ativa			0,518*
Não	45 (42,8)	28 (26,6)	
Sim	17 (16,1)	15 (14,2)	
Radioterapia ativa			0,714*
Não	58 (55,2)	39 (37,1)	
Sim	4 (3,8)	4 (3,81)	
Cirurgia recente			0,661*
Não	46 (43,8)	30 (28,5)	
Sim	16 (15,2)	13 (12,3)	

Resultados de idade em mediana e variação, demais variáveis expressas como números absolutos e percentual.

* Teste do χ^2^; ^†^ teste *t* de Student.

DM: *diabetes mellitus*; ICC: insuficiência cardíaca congestiva; ECOG: *Eastern Cooperative Oncology Group*.

O câncer de mama foi o tipo de mais frequente, seguido por câncer ginecológico e gastroinsteinal (38/36,1%, 35/33,3% e 9/8,57% pacientes afetados, respectivamente). Sessenta pacientes (57%) tinham doença estágio IV, e 40% apresentavam bom estado de desempenho, de acordo com o ECOG 0-1, no momento do diagnóstico de COVID-19.

Dos 60 pacientes (30,4%) que estavam sob terapia anticâncer ativa, 42 (70%) receberam quimioterapia citotóxica, seis (10%) terapia-alvo e 12 (20%) terapia endócrina. Nenhum dos pacientes incluídos nesta amostra recebeu terapia com bloqueio de *checkpoint* imunológico. Oito pacientes (7,6%) estavam sendo tratados com radioterapia, e 29 tinham sido operados recentemente (isto é, 30 dias ou menos antes dos primeiros sintomas de COVID-19). Não foram observadas associações entre terapia anticâncer ativa (cirurgia, radioterapia ou tratamento sistêmico) e mortalidade.

As principais comorbidades encontradas entre os pacientes com COVID-19 foram hipertensão (38%), diabetes (20,9%) e doença cardíaca (6,7%). A dispneia, a tosse e a febre foram os principais sintomas apresentados no momento do diagnóstico de COVID-19 45,6%, 38% e 30,4% dos pacientes, respectivamente. Outros sintomas foram fadiga (13,3%), mialgia (13,2%), e anosmia (5,7%).

### Parâmetros clínicos e desfechos dos pacientes com câncer e COVID-19

Em 5 de julho de 2020, 49 de 105 (46,6%) pacientes com câncer e COVID-19 foram considerados em recuperação ou curados (acompanhamento médio de 30 dias). Faleceram 43 pacientes (40,92%) por ocasião do último acompanhamento (Tabela 2).

**Tabela 2:** Parâmetros clínicos e desfechos dos pacientes com câncer e COVID-19

Variável	Total (%)	Vivos (%)	Falecidos (%)	Valor de p
Internação, n (%)	73 (69,5)	30 (28,5)	43 (40,9)	0,001
Tempo de internação hospitalar, dias	17,9±12,9	14,7±11,7	20,1±13,3	0,03
Internação na UTI, n (%)	35 (33)	4 (3,8)	31 (29,5)	0,001
Tempo de internação na UTI, dias	4,7±6,8	0,5±2,2	8,4±7,4	0,001
Ventilação mecânica, n (%)	34 (32,3)	2 (1,9)	32 (30,4)	0,001
Coagulação intravascular disseminada, n (%)	3 (2,86)	0	3 (2,86)	0,001
Pneumonia, n (%)	49 (46,6)	14 (13,3)	35 (33,3)	0,001
Terapia de substituição renal, n (%)	25 (23,8)	3 (2,8)	22 (20,9)	0,001
Choque séptico, n (%)	31 (29,5)	1 (0,9)	30 (28,5)	0,001

Resultados expressos por números absolutos e percentual ou média±desvio-padrão.

UTI: unidade de terapia intensiva.

Setenta e três pacientes (69,5%) necessitaram de internação hospitalar devido à COVID-19, e 32 (30,4%) tiveram alta. O tempo de internação hospitalar variou de 12,9 a 17,9 dias.

A taxa de mortalidade dos pacientes que necessitaram de internação hospitalar foi de 40,95%, sendo mais elevada entre os pacientes do sexo masculino (apenas 20% de sobrevida até o tempo de corte adotado) do que entre os do sexo feminino (68,2% de sobrevida). Após a internação hospitalar, 35 pacientes (33,3%) precisaram ser transferidos para a UTI. Destes, apenas quatro tinham sobrevivido até a data do último acompanhamento.

Os pacientes hospitalizados foram submetidos a exames laboratoriais (hemograma, bioquímica sérica, teste de coagulação, testes de função hepática e renal, dosagem de eletrólitos, proteína C-reativa, procalcitonina, D-dímero e lactato desidrogenase). Os marcadores laboratoriais não foram preditores de maior risco de mortalidade.

Neste estudo, os pacientes que necessitaram de ventilação mecânica ou terapia de substituição renal tenderam a apresentar taxa mais alta de mortalidade (32 de 34 pacientes/94,1%, e 22 de 25/88%, respectivamente). A dispneia foi o sintoma inicial mais prevalente entre os pacientes que faleceram (30,4%) e mostrou-se significativamente (p<0,0001) associada a esse desfecho.

A internação hospitalar, a necessidade de internação da UTI e a permanência mais longa na UTI tiveram impacto negativo significativo (p<0,0001) sobre o desfecho dos pacientes, mas não o tempo de internação hospitalar (p=0,03).

Cinco casos de arritmias (um bloqueio de ramo direito e quatro fibrilações atriais com alta resposta ventricular) e três eventos microvasculares trombóticos (coagulação intravascular disseminada) foram identificados nesta amostra. Os pacientes que desenvolveram arritmia ou coagulação intravascular disseminada evoluíram para morte. Não foram registrados eventos dermatológicos, uma vez que as associações entre tais eventos e a COVID-19 já tinham sido reconhecidas quando o registro foi criado.

### Análise de regressão logística e chances de morte, de acordo com as características clínicas dos pacientes

Neste estudo, *performance status* ruim (razão de chance – RC de 5,15; intervalo de confiança de 95% – IC95%: 1,15-22,97; p=0,031) e sexo masculino (RC de 7,29; IC95%: 1,01-52,33; p=0,048) mostraram-se associados ao maior risco de morte (análise de regressão logística múltipla demonstrada na tabela 3). A ordem de não ressuscitar e a doença em estágio IV (presença de metástase) não se mostraram associadas à maior mortalidade.

**Tabela 3:** Análise de regressão logística e chances de morte, de acordo com as características clínicas dos pacientes

Características	RC (IC95%)	Valor de p
Sexo	7,29 (1,01-52,33)	0,048
Idade	1,01 (0,96-1,06)	0,594
*Performance status*	5,15 (1,15-22,97)	0,031
Ordem de não ressuscitação	0,48 (0,08-2,82)	0,419
Proteína C-reativa (mg/dL)	1,02 (0,95-1,09)	0,482
Doença estágio IV	3,06 (0,66-19,25)	0,139
Número de comorbidades	0,77 (0,30-1,95)	0,590

RC: razão de chance; IC95%: intervalo de confiança de 95%.

### Análise exploratória dos pacientes, de acordo com o escore de *performance status*

Além dos fatores listados na tabela 4, a análise exploratória de acordo com o escore de *performance status* desta população de pacientes com COVID-19 revelou associações significantes (p<0,0001) entre a gravidade da COVID-19 e a internação na UTI, a necessidade de ventilação mecânica e o maior tempo de permanência na UTI, mas não com a duração da internação (p=0,059).

**Tabela 4:** Análise exploratória dos pacientes, de acordo com o escore de *performance status*

Desfechos clínicos	ECOG 0-1 (n=42)	ECOG 2-4 (n=62)	Valor de p
Necessidade de internação hospitalar, n (%)	18 (17,3)	54 (51,9)	<0,001
Tempo de internação hospitalar, dias	11±6,9	19,9±13,6	0,059
Internação na UTI, n (%)	7 (6,3)	27 (25,9)	0,005
Tempo de internação na UTI, dias	2,0±3,9	5,56±7,32	0,007
Ventilação mecânica, n (%)	7 (6,3)	26 (25)	0,005
Óbito, n (%)	7 (6,73)	35 (33,6)	<0,001

Resultados expressos por números absolutos e percentual ou média±desvio-padrão.

ECOG: *Eastern Cooperative Oncologic Group*; DP; desvio padrão; UTI: unidade de terapia intensiva.

## DISCUSSÃO

O surto de COVID-19 se transformou em uma pandemia viral temida por pacientes com câncer e equipes de oncologia, dados os impactos negativos importantes no tratamento do câncer.^(15)^ Esta pesquisa descreve as características clínicas e epidemiológicas de pacientes com câncer e COVID-19 atendidos em um centro brasileiro de oncologia.

O IBCC Oncologia é um centro de referência no Brasil. Aproximadamente 15 mil pacientes foram tratados de forma ativa no IBCC Oncologia nos 3 meses que antecederam o surto de COVID-19. Destes, apenas 105 foram diagnosticados com infecção por SARS-CoV-2, entre 16 de março e 29 de maio de 2020. Estima-se que 170.342 pessoas que residem na área urbana de São Paulo, (SP) tenham sido infectadas pelo SARSCoV-2 até a data deste relato.^(16)^

A incidência de COVID-19 em pacientes com câncer não pode ser estimada com base neste estudo. A incidência aparentemente baixa pode ter refletido a adoção de medidas preventivas, como o distanciamento social por parte desses pacientes, que tinham sido devidamente informados do risco potencialmente mais alto de COVID-19 grave, ou as medidas profiláticas implementadas no IBCC Oncologia para garantir um fluxo seguro de pacientes no hospital, conforme proposto em diversas diretrizes.^(17-21)^

O câncer de mama e o ginecológico foram os tipos mais comuns de neoplasias nessa coorte. Achados semelhantes foram relatados em estudos prévios que descreveram relações entre tipo de câncer e COVID-19.^(22)^ Diferentemente de outros estudos, a alta prevalência de câncer pulmonar não foi observada nesta amostra.^(23)^ Essa discrepância pode ser explicada pelo fato de o IBCC Oncologia ser um centro especializados em tumores em mulheres.

Os sintomas típicos de COVID-19 observados nos pacientes com câncer neste estudo (febre, tosse, dispneia, fadiga, mialgia e anosmia) não diferiram dos relatados na população em geral.^(24)^

Nesta coorte, a dispneia foi o primeiro sintoma vivenciado pelos pacientes que evoluíram para óbito (30,4%), e ambos os eventos se mostraram significativamente (p<0,0001) associados. Esse achado indica que a dispneia requer atenção especial no tratamento de pacientes com câncer e COVID-19.

De acordo com estudos prévios, a dispneia, em pacientes com câncer de pulmão, manifesta-se em estágio muito mais precoce, logo no início da COVID-19, comparativamente à população em geral e aos pacientes que sofrem de outros tipos de câncer. Pacientes com câncer de pulmão, por apresentarem comprometimento da função pulmonar e da resistência pulmonar já no início da doença, são mais predispostos ao desenvolvimento de anóxia grave e tendem a apresentar COVID-19 de evolução rápida.^(24,25)^ O baixo número de casos de câncer pulmonar nesta amostra (dois) impossibilitou a análise desses fatores na amostra.

A taxa de mortalidade dos pacientes que necessitaram de internação hospitalar nessa coorte (40,95%) é compatível com os achados de revisão sistemática de 52 estudos envolvendo 18.650 pacientes com câncer e COVID-19, uma indicação clara de que a mortalidade é, de fato, mais alta entre esses pacientes, e a internação hospitalar deve ser considerada um fator de risco independente.^(26)^ A análise de regressão logística revelou dois fatores de risco independentes para mortalidade neste estudo: sexo masculino e estado de *performance status* ECOG 2 ou superior. A mortalidade mais alta entre os pacientes do sexo masculino pode ter refletido o pequeno número de homens nesta amostra. Além disso, dado o pequeno tamanho amostral, o *performance status* desfavorável pode ter compensado outros fatores de risco.

Contrastando com os achados deste relato, diversos outros estudos realizados ao longo dos meses de vigência da pandemia não apontaram taxas mais altas de mortalidade entre pacientes com câncer e COVID-19.^(27-40)^

Os dados apresentados neste relato sugerem que pacientes com câncer e escore de ECOG *performance status* igual ou superior a dois devem ser encarados como uma população de extrema vulnerabilidade, que deve ser alvo prioritário de medidas de isolamento social.

Estudo brasileiro de coorte com pacientes com câncer e COVID-19, realizado por de Melo et al., e publicado em outubro de 2020, revelou achados semelhantes. A taxa de mortalidade específica para COVID-19 em pacientes com tumores sólidos foi de 37,7%, e as taxas de complicações, como insuficiência respiratória, choque séptico e lesão renal aguda, foram bastante elevadas (38,7%, 22,1% e 18,2%, respectivamente).^(41)^

Este estudo tem algumas limitações. Em primeiro lugar, no início da pandemia de COVID-19, os pacientes buscavam cuidados médicos somente em casos de sintomas graves, como dispneia, febre persistente ou dor torácica. Portanto, os casos graves de COVID-19 podem ter sido super-representados, o que pode, em parte, explicar o alto índice de internação hospitalar nesta amostra (70%). Em segundo lugar, os pacientes com sintomas leves de COVID-19 podem ter optado por ficar em casa ou buscar atendimento médico em unidades de Atenção Primária de Saúde.

Esta análise foi baseada nos primeiros casos de COVID-19 registrados no IBCC Oncologia. Estudos futuros devem ser realizados, dados o número crescente de casos.

## CONCLUSÃO

Apesar da adoção de medidas preventivas de distanciamento social por parte de pacientes com câncer e das medidas profiláticas implementadas no Instituto Brasileiro de Controle do Câncer para garantir o fluxo seguro de pacientes no hospital, esta análise revelou altas taxas mortalidade por COVID-19 entre pacientes com câncer do sexo masculino e em pacientes com escore de *performance status* ruim.

Tais achados destacam a vulnerabilidade dos pacientes com câncer na pandemia vigente e podem contribuir para uma seleção mais adequada de pacientes, que podem se beneficiar de medidas rígidas de isolamento e, eventualmente, da interrupção do tratamento antineoplásico, a fim de reduzir a exposição à infecção por COVID-19.
